# Biodiversity and Spatiotemporal Variations of Mecoptera in Thailand: Influences of Elevation and Climatic Factors

**DOI:** 10.3390/insects15030151

**Published:** 2024-02-23

**Authors:** Theerapan Dokjan, Wesley J. Bicha, Piyawan Suttiprapan, Bajaree Chuttong, Chun-I. Chiu, Kittipat Aupalee, Atiporn Saeung, Chayanit Sulin, Wichai Srisuka

**Affiliations:** 1 Department of Entomology and Plant Pathology, Faculty of Agriculture, Chiang Mai University, Chiang Mai 50200, Thailand; theerapan_d@cmu.ac.th (T.D.); bajaree.c@cmu.ac.th (B.C.); chuni.chiu@cmu.ac.th (C.-I.C.); 2 Independent Researcher, 121 Old Batley Road, Oliver Springs, TN 37840, USA; mecoptera@live.com; 3 Parasitology and Entomology Research Cluster (PERC), Department of Parasitology, Faculty of Medicine, Chiang Mai University, Chiang Mai 50200, Thailand; kittipat.aupalee@gmail.com; 4 Entomology Section, Queen Sirikit Botanic Garden, Chiang Mai 50180, Thailand; chayanitsulin@gmail.com (C.S.); wsrisuka@gmail.com (W.S.)

**Keywords:** Bittacidae, hangingfly, Indochina, insect, Malaise trap, pan trap, Panorpidae, Oriental region, scorpionfly

## Abstract

**Simple Summary:**

Mecoptera, commonly known as scorpionflies and hangingflies, are an interesting, but small, and rarely-observed order of moderately-sized insects, with at least 22 species in Thailand. These insects typically prefer cool, moist mature forested habitats with broken sunlight, often on low vegetation bordering small mountain streams. They are poor fliers thus limiting their ability to disperse. For these reasons, they serve as a valuable bioindicator for monitoring changes in climatic and forest communities. However, no ecological study has previously been conducted for this insect group. This study exploits data from Project Tiger, where specimens were collected using Malaise and pan traps from 18 national parks across Thailand to characterize the biodiversity of Mecoptera with a focus on the influences of elevational and climatic factors. We found 21 species in total with 52% of them being specific to particular regions. Negative correlations between species richness and abundance of Mecoptera with both elevation and temperature were observed, suggesting a predominance of species in mid-altitude areas with a peak in the rainy season (21 species) followed by the hot (10 species) and cold (7 species) seasons. We suggest that the narrow distribution and preference for mid-altitude make Mecoptera particularly vulnerable to global warming, raising urgent conservation concerns.

**Abstract:**

Ecological analyses of the small and lesser-known insect order Mecoptera in Thailand are presented. Specimens were collected monthly over a period of 12 consecutive months, using both Malaise and pan traps, from 29 sampling sites located in 18 national parks throughout Thailand. A total of 21 species in four genera were identified from 797 specimens, including *Panorpa* (1 species), *Neopanorpa* (18 species), *Bittacus* (1 species), and *Terrobittacus* (1 species), with the latter genus representing a new genus record to Thailand. *Neopanorpa harmandi*, *N. siamensis*, *N. byersi*, and *N. malaisei* were the most abundant species, representing 27.4%, 11.3%, 10.3% and 8.8% of the total specimens, respectively. The species with the highest frequency, as indicated by the high percentage of species occurrence (%SO), was *N. siamensis* (51%), followed by *N. byersi* (34%), *N. harmandi* (34%), *N. spatulata* (27%), and *N. inchoata* (27%). Eleven species (52%) exhibited specific regional occurrences. *N. tuberosa* and *N. siamensis* had the widest distribution, being found in almost all regions except for western and southern regions for the first and second species, respectively. The seasonal species richness of Mecoptera was high during the rainy season in the northern, northeastern, central, eastern, and western regions, with the highest richness observed in July (15 species), followed by the hot (10 species) and cold seasons (7 species), while there was no significant difference in species richness between seasons in the southern region. Multiple regression models revealed a negative association between species richness and abundance of Mecoptera with both elevation and temperature, and a positive association between rainfall and species evenness. It is predicted that climatic changes will have a detrimental effect on the mecopteran community. The results of this study enhance the understanding of the ecological aspects of Mecoptera, offering crucial insights into its biodiversity and distribution, which are vital for conservation and forest management.

## 1. Introduction

Mecoptera are commonly referred to as “Scorpionflies”, a name which characterizes the adult males of the Panorpidae, whose genitalia are bulb-shaped, and their upturned abdominal tips resemble the stingers of scorpions. Adults of the Bittacidae typically hang from the undersides of herbaceous plants or shrubs, acting as predators that capture small insects with their raptorial hind tarsi, and are often called “Hangingflies”. Despite these differences, Mecoptera are readily characterized by their mouthparts, which extend into an elongated rostrum, and their fore and hind wings that are long, of similar size, and share a similar venation pattern [1,2,3]. Mecoptera comprise 40 genera within nine families and have relatively low species diversity, with approximately 800 species distributed globally. A substantial majority (91%) of this insect order is found in the Northern Hemisphere, with only a small percentage occurring in the Southern Hemisphere [2,3,4,5,6]. Among the nine families, Panorpidae and Bittacidae are the two largest, accounting for 90% of the total species [7,8]. To date, only the families, Panorpidae and Bittacidae, with 43 and 5 species, respectively, are present in the Oriental region, suggesting that this region serves as a biodiversity hotspot for Mecoptera [2,5,9].

In forest ecosystems, Mecoptera exhibit a diverse range of nutritional sources, such as decaying animals, vegetative materials, and other soft-bodied organisms, which support their vital role in recycling carrion within ecosystems, especially in mountainous areas [10]. Most intriguingly, panorpids may have value in forensic entomology, as they have been observed to arrive at human cadavers in less than 20 min [2,11]. Panorpids have also been known to consume pollen, fermenting berries, feces, and nectar, and even exhibit phytophagous behavior [3,8,10,12,13,14,15,16,17]. Hangingflies, on the other hand, are predatory and target small arthropods such as dipterans, lepidopterous larvae, and adults of Hemiptera and Hymenoptera [2,18]. Some *Panorpa* species display kleptoparasitism by accessing the webs of web-building spiders to feed on ensnared insects [19,20]. It is evident that their ancestors, possessing a long proboscis and feeding on gymnosperm pollen, may have served as early plant pollinators during the Late Jurassic to Early Cretaceous periods [2,21].

Mecoptera, especially “panorpids”, exhibit specific habitat preferences, predominantly favoring mountainous areas characterized by high moisture levels. Adult panorpids are found commonly resting on leaf surfaces, while bittacids hang within the vegetation [3,9,14,18]. Due to their constrained migration capabilities, restricted distribution, preference for lower temperatures, and sensitivity to climate change and vegetation shifts, Mecoptera serve as invaluable models for studying the impacts of climate change and deforestation. Furthermore, they play a significant role in insect conservation by functioning as ecological indicators [10,14,17,22,23]. Thus, gaining insights into insect biodiversity, spatiotemporal variations, and the influences of environmental factors remain a paramount ecological challenge [24,25,26]. Certain ecological studies on Mecoptera have highlighted the pivotal roles of precipitation and elevation in determining species assemblages. They indicate that the most suitable habitats for scorpionfly distribution often exhibit a mid-elevational peak or hump-shaped pattern in species richness [10,17,22]. As a result, continuous surveys and research on species–environment relationships in distinct habitats, or previously unexplored regions, are essential for deepening the understanding of biodiversity, and enhancing conservation efforts for Mecoptera.

Thailand is ideally situated in the Oriental region and boasts a unique geographical position. It encompasses a transitional zone from the temperate, mountainous fringes of continental Asia in the north to the dense rainforests of the Malayan peninsula in the south. This placement endows Thailand with rich biodiversity, particularly in its flora and fauna [27]. Insects are especially diverse, with over 10,000 species recorded [28], and numerous studies have reported on insect diversity, distribution, and community structure within the country [29,30,31,32,33]. In the case of Mecoptera, there has been a significant increase in the number of species identified in Thailand over the past five years. The initially reported 11 species has expanded to 22 described species, comprising 2 *Panorpa*, 19 *Neopanorpa*, and 1 *Bittacus* species. We are aware of at least an additional undescribed *Panorpa* and a *Terrobittacus*. This surge was due mainly to the second author’s reports on new species and records of scorpionflies, derived from specimens available through the Thailand Inventory Group for Entomology Research (TIGER project) and additional surveys by W.S. and W.J.B. This initiative has greatly influenced insect taxonomic work in Thailand, resulting in the publication of over a hundred papers spanning various insect groups [5]. While the taxonomic study of Mecoptera in Thailand has seen swift advancements, ecological studies are sparse. This research marks the inaugural effort to report on the biodiversity and distribution of Mecoptera across the country.

While Bicha (2019) [5] has provided some insights into the spatial and temporal distribution of Mecoptera in Thailand, the data remain limited. In addressing this gap and understanding the impacts of climatic factors, this study examined the diversity of Mecoptera in 29 sites located in 18 national parks of Thailand, and analyzed their community structure, spatiotemporal variations, regional distribution, and correlations with climatic factors.

## 2. Materials and Methods

### 2.1. Study Sites

**Figure 1:**
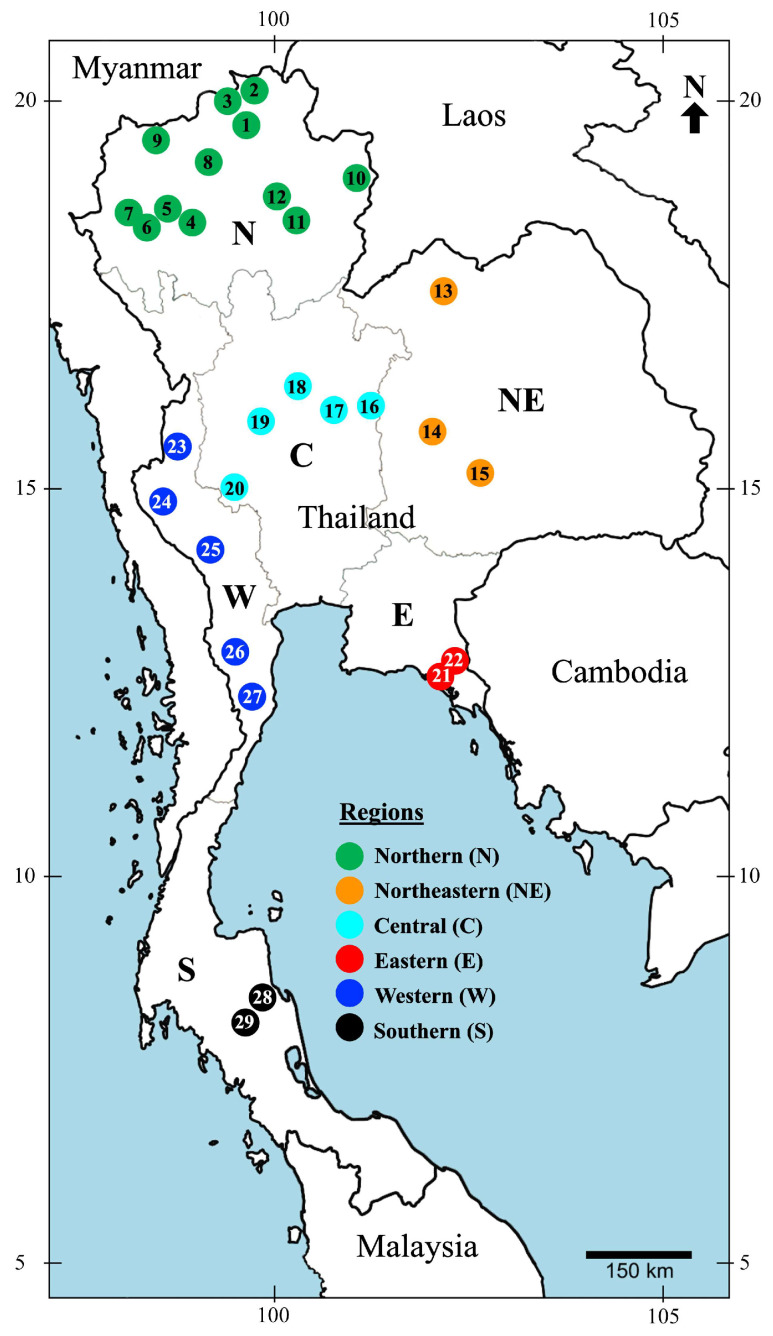
Map of Thailand showing 29 collection sites across six regions. Detailed locality information is in Table S1.

Mecopteran specimens were collected from 29 collection sites in 18 national parks across six regions of Thailand, which are as follows: northern (12 sites), northeastern (3 sites), central (5 sites), eastern (2 sites), western (5 sites), and southern (2 sites), as depicted in Figure 1. The collections were conducted under permit numbers 0002.3/5075 (from 2006 to 2009) and 0907.4/20861 (from 2020 to 2021), both of which were approved by the Department of National Park, Wildlife, and Plant Conservation. The seasonal classification and regional demarcation were based on the criteria established by the Thai Meteorological Department [34]. This classification divides the year into three distinct seasons, based on variations in rainfall and air temperature: rainy (May–October), dry cold (November–January), and dry hot (February–April). This applies to the northern, central, northeastern, eastern, and western regions. Conversely, the southern region is divided into two seasons: hot (November–April) and rainy (May–October).

### 2.2. Collection Methods

Mecopteran specimens for this study were collected using both Malaise and pan traps. The Malaise trap, measuring 100 cm in width, 170 cm in length, and 150 cm in height, was equipped with a 500 mL collection bottle containing 85% ethanol and glycerine. These traps were deployed at each collection site for 12 consecutive months and serviced monthly. The pan trap, with a diameter of 23 cm and height of 3 cm, was filled with water and a few drops of surfactant (dish-washing liquid). Five pan traps were placed at each collection site, at 5 m intervals and operated for 5 consecutive days, requiring daily servicing. From each sample, mecopteran specimens were separated from other insects by morphological differentiation in the laboratory. They were then counted and preserved individually in 5 mL plastic tubes containing 80% ethyl alcohol.

### 2.3. Species Identification

Species identification was carried out morphologically under a stereomicroscope, utilizing a key specific to Mecoptera of Thailand [3,5,35,36]. This was supplemented by additional publications on Mecoptera in Thailand and its neighboring countries [37,38]. Representative specimens from this study have been deposited at the Entomology Section of the Queen Sirikit Botanic Garden (QSBGE) in Chiang Mai, Thailand.

### 2.4. Climatic Variables

Climatic variables, comprising monthly minimum temperature, maximum temperature, and precipitation, were extracted from WorldClim’s “Historical monthly weather data” for the decade spanning 2006–2009 (4 years) [39]. These climatic datasets were converted subsequently to raster format utilizing the “raster” package in R. Furthermore, the climatic variables corresponding to each collection site were ascertained using the “extract” function within the R programming environment [40]. To consolidate and obtain the mean values of climatic variables over a four-year period, the following metrics were computed: average annual precipitation (AAP), average monthly warmest temperature (AMWT), and average monthly coolest temperature (AMCT). Elevation of each collection site was obtained through a global positioning system (GPS) recorder.

### 2.5. Statistical Analyses

In order to elucidate the community structure of Mecoptera across various collection sites, species richness indices, including Shannon (H′), Simpson (1-D), and evenness (e^H/S), were calculated at each collection site. Chao1 richness estimator was calculated in each national park. Furthermore, the relative abundance (RA) and percentage of species occurrence (%SO) of each species were determined, based on samples aggregated from all of the collection sites. The RA was computed using the formula, RA = ni/N × 100, where “ni” represents the total count of specimens for species “I”, and “N” denotes the overall number of specimens gathered, as described by [41]. The %SO represents proportion of the number of collections in which a species is present, relative to the total number of collections (*n* = 29).

A contour map was generated using a matrix plot to visualize variations in elevational and monthly abundance of mecopteran species. To explore the potential association between species composition and geographical regions in Thailand, the Bray–Curtis similarity index was computed based on abundance data of mecopteran species assemblages. Cluster analyses using the unweighted pair group method with arithmetic mean (UPGMA) were conducted, supported by 1000 bootstrap replications. Samples obtained from the Malaise and pan traps were combined for the analyses. To assess effects of the collection methods (Malaise and pan traps) on species diversity, the diversity *t*-test was performed using the diversity index (Shannon_H index). Additionally, the two-sample *t*-test was used to compare mean abundance, and the species abundance distribution was characterized using the log-series model. All previously mentioned analyses were conducted using PAST version 4.08 [42].

Multiple regression models were computed by using R function lm, to analyze the associations between species richness, abundance, evenness indices and environmental factors, namely elevation, average annual precipitation (AAP), average monthly warmest temperature (AMWT), and average monthly coolest temperature (AMCT). The species accumulation curves of rarefaction and extrapolation were also generated, based on varied samples (abundance data), using the iNEXT package in R for diversity comparisons across collection methods, seasons, and elevations. To ensure sampling adequacy, analyses were conducted with 1000 randomizations without replacement [43,44]. All R packages and functions utilized in this study were implemented within the R programming environment, version 4.3.0 [40].

## 3. Results

### 3.1. Biodiversity and Community Structure of Mecoptera in Thailand

#### 3.1.1. Biodiversity of Mecoptera in Thailand

A total of 797 mecopteran specimens representing 21 species of 4 genera were collected including *Bittacus* (1 species), *Neopanorpa* (18 species), *Panorpa* (1 species), and *Terrobittacus* (1 species). This is the inaugural report of hangingflies belonging to the genus *Terrobittacus*, marking it as a novel genus record for Thailand. One male was discovered at Mae Klong Kee, and four females at Thi Lor Su, both located in Umphang, Tak province (Table 1).

**Figure 2:**
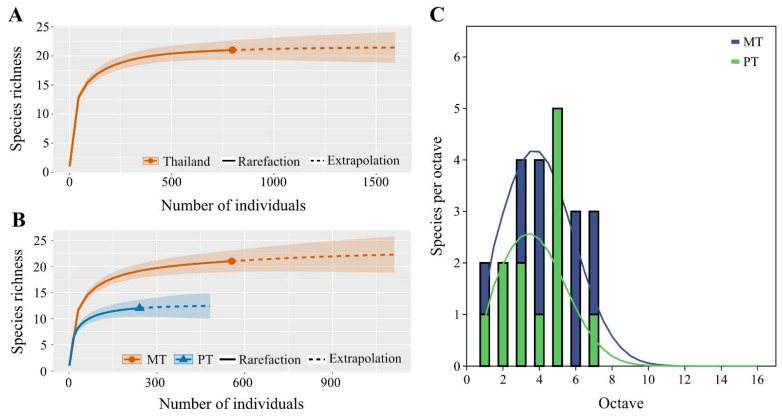
Evaluation of sampling effort for estimating the overall species diversity of Mecoptera. Species accumulation curves (sample rarefaction based on abundance data) exhibit asymptotic behavior in two scenarios: (**A**) across all samples, and (**B**) within samples gathered using both Malaise trap (MT) and pan trap (PT) collection methods. In panels A and B, the 95% confidence intervals are depicted by the shaded area. The solid line represents the observed species richness (interpolation), whereas the dashed line indicates the extrapolated species richness. The sample coverage for the reference sample is noted as 0.998, with q = 0, and the species richness is calculated based on 1000 bootstrap samples. A higher diversity is evident in the samples collected via the MT method (**B**). (**C**) Log scale plot of abundance distribution of mecopteran specimens collected by the Malaise trap (MT) and pan trap (PT).

The highest diversity index was observed at Doi Pha Luang, followed by Thi Lor Su, and the Headquarters of Khao Yai National Park, with values of 1.58, 1.52 and 1.45, respectively. Diversity information for other collection sites is presented in Table S1. A total of 21 species were observed in this study, whereas the Chao-1 estimated the total species richness in Thailand at 72 species (Table S1). Asymptotic behavior of species accumulation curves (sample-based rarefaction) suggested that the sampling efforts in this study were sufficient (Figure 2A).

A diversity comparison between the collection methods was analyzed. Malaise and pan traps showed divergent results in species diversity and abundance of Mecoptera. The species accumulation curve suggested a higher capture rate with Malaise traps (21 species) compared to pan traps (12 species) (Figure 2B). The diversity *t*-test supported this, revealing a significant difference in the diversity (Shannon_H index) between the methods (t = 8.02, df = 419.22, *p* < 1.0721 × 10^−14^), with Malaise traps recording a higher index (H = 2.5) than pan traps (H = 1.9). Moreover, a significant difference in mean abundance was also noted (t = 3.55, df = 20, *p* < 0.0021) as in Figure 2C, revealing a lognormal distribution for each method. Notably, the distribution from the Malaise trap demonstrates a higher peak, indicating greater abundance.

#### 3.1.2. Community Structure of Mecoptera in Thailand

Among 21 mecopteran species discovered in this study, *Neopanorpa harmandi*, *N. siamensis*, *N. byersi* and *N. malaisei* were the four most relatively abundant species, comprising 27.4%, 11.3%, 10.3% and 8.8% of the total collected specimens, respectively (Table 1).

Five species were the most prevalent, as determined by their %SO. *N. siamensis* led with 51.7%, followed by *N. byersi* and *N. harmandi* (both at 34.5%), *N. spatulata* (27.6%), and *N. inchoata* (27.6%). These species were found in 15, 10, 10, 8, and 8 collection sites, respectively. Conversely, six and five species of Mecoptera were found in only one and two collection sites respectively, as detailed in Table 1.

The number of species in each collection site ranged from one to seven. A majority of the sites (25 out of 29, or 86.2%) collected between one and five species, with four of those collecting only one. Conversely, 4 out of the 29 sites, or 13.8% of the total, showcased a diversity of 6–7 species. The richest species diversities were observed in Doi Phaluang and Thi Lor Su, both presenting seven species. This was followed by Pha Tan substation and Kiew Lom, each with six species. The species distribution in other collection sites is detailed in Table 1.

The species composition and abundance of Mecoptera in 18 national parks varied, with the number of species ranging from 1 to 12. The highest species diversity was observed in Doi Pha Hom Pok (12 species), followed by Umphang (8 species), Doi Inthanon (7 species), and Doi Chiang Dao (6 species). In contrast, three national parks (Khao Kho, Mae Wong, and Khao Khitchakut) each exhibited only two species, while Khuean Srinagarindra National Park had just one species. Notably, Doi Pha Hom Pok was the most diverse among them, hosting 12 species in two genera. *Neopanorpa arcuata* was the most abundant species in this park, accounting for 24% of the total specimens collected, followed by *N. inchoata* and *N. malaisei*, at 15% and 14%, respectively. On the other hand, *N. cuspidata* was the least abundant (0.8%). The composition of mecopteran species and their abundance in other national parks are presented in Table 1.

#### 3.1.3. Regional Distribution of Mecoptera in Thailand

**Figure 3:**
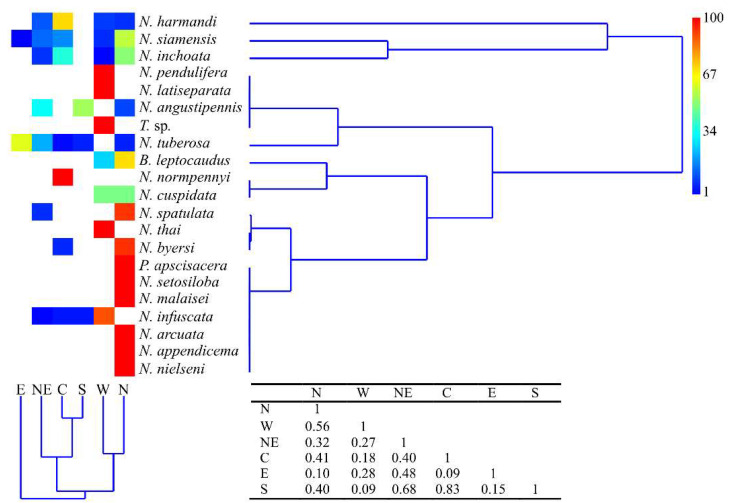
Cluster analysis using the Bray–Curtis resemblance coefficient and UPGMA to produce the dendrogram (Copen. Corr = 0.97), based on species distribution (abundance data) in the six regions of Thailand. The numbers in the table indicate the Bray–Curtis similarity index between regions. Abbreviations of geographical regions are as follows: N = northern, NE = northeastern, C = central, E = eastern, W= western and S = southern.

Regional similarities based on the abundance data of species distribution, as determined by multivariate analysis using the Bray–Curtis similarity index, indicated that the mecopteran community exhibited high similarity between the central and southern regions (83%), northeastern and southern regions (68%), and western and northern regions (56%), while the lowest similarity was observed between western and southern regions (9%) (Figure 3).

Interestingly, 52% or 11 out of the 21 species had region-specific distribution. The data indicate that the majority of species (71%, 15 out of 21 species) were distributed in the northern region, with six species restricted entirely to this region (*Neopanorpa appendicema*, *N. arcuata*, *N. malaisei*, *N. nielseni*, *N. setosiloba* and *Panorpa apscisacera*). Ten species were found in the western region, including four specific ones (*N. latiseparata*, *N. pendulifera*, *N. thai* and *Terrobittacus* sp.). Seven species were recorded in the central region, in which *N. normpennyi* occurred exclusively. Notably, *N. siamensis* and *N. tuberosa* were found to have the widest distribution range, being found in five out of the six regions, excluding the southern and western region for the former and latter, respectively. *N. siamensis* showed the highest abundance in the northern region, while *N. tuberosa* was the most abundant in the eastern region. Three other species, *N. harmandi*, *N. inchoata*, and *N. infuscata* were distributed in four out of the six regions, with specific regions varying depending on the species (Figure 3).

### 3.2. Spatiotemporal Variation of Mecoptera in Thailand

#### 3.2.1. Seasonal Species Richness and Monthly Variation or Emergence Time

**Figure 4:**
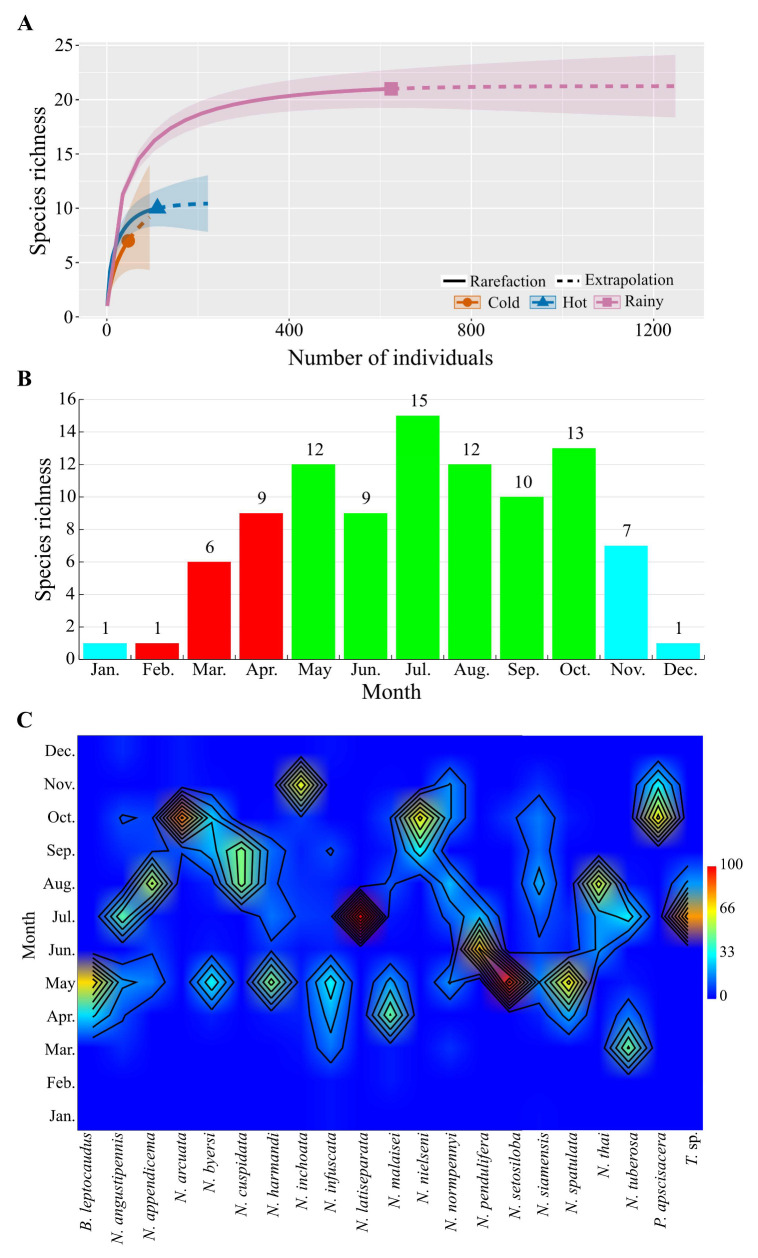
Species richness variations of Mecoptera in Thailand (except for the southern region). (**A**) Seasonal species richness comparison of Mecoptera in Thailand. The shaded area represents 95% confidence intervals. Solid line = observed species (interpolation); dashed line = extrapolated species richness. Sample coverage of the reference sample for each season: cold season = 0.937; hot season = 0.999 and rainy season = 0.998, q = 0, species richness with 1000 bootstrap samples. (**B**) Monthly variation in species richness (red bar = hot season, green bar = rainy season, and aqua bar = cold season). (**C**) Monthly specific abundance (%) of mecopteran species.

The species accumulation curve clearly revealed differences in species richness between the seasons, with a peak in the rainy season (21 species), followed by the hot (10 species) and cold seasons (7 species) (Figure 4A). In the southern region, both species richness and abundance were similar across seasons, with the same number of two species found in both the rainy and hot seasons, although these species were different. In total, three species were found in this region: *N. angustipennis* (12 individuals), *N. infuscata* (2 individuals), and *N. tuberosa* (2 individuals). *N. angustipennis* was present in both seasons, with a higher abundance in the rainy season (7 individuals), whereas *N. infuscata* was found exclusively in the hot season, and *N. tuberosa* only in the rainy season.

The monthly variation in species richness of Mecoptera in Thailand is presented in Figure 4B. With exception of the southern region, species richness peaked in July (15 species), followed by October (13 species), May (12 species) and August (12 species), while it reached its lowest point in January, February, and December.

In terms of seasonal variation, the rainy season is most suitable for Mecoptera, with 18 species displaying high abundance during that period. Most of these species reached their peak in May and August, while a minority dominated during the hot (two species) and cold seasons (one species). Seven species occurred in all seasons, including *Neopanorpa angustipennis*, *N. byersi*, *N. infuscata*, *N. normpennyi*, *N. siamensis*, *N. inchoata*, and *N. malaisei*. Apart from the last two species, the remainder exhibited their highest abundance during the rainy season. Eight species were restricted only to the rainy season, namely *N. appendicema*, *N. cuspidata*, *N. latiseparata*, *N. nielseni*, *N. pendulifera*, *N. setosiloba*, *N. thai*, and *Terrobittacus* sp. In contrast, four species, *Bittacus leptocaudus*, *N. harmandi*, *N. spatulata*, and *N. tuberosa*, occurred during both the rainy and hot seasons, with the first three species reaching their peak abundance in the rainy season, and the last one during the hot season. Additionally, two species, *N. arcuata* and *Panorpa apscisacera*, were found in the transition between the rainy and cold seasons (Figure 4C).

#### 3.2.2. Elevational Species Richness and Abundance

**Figure 5:**
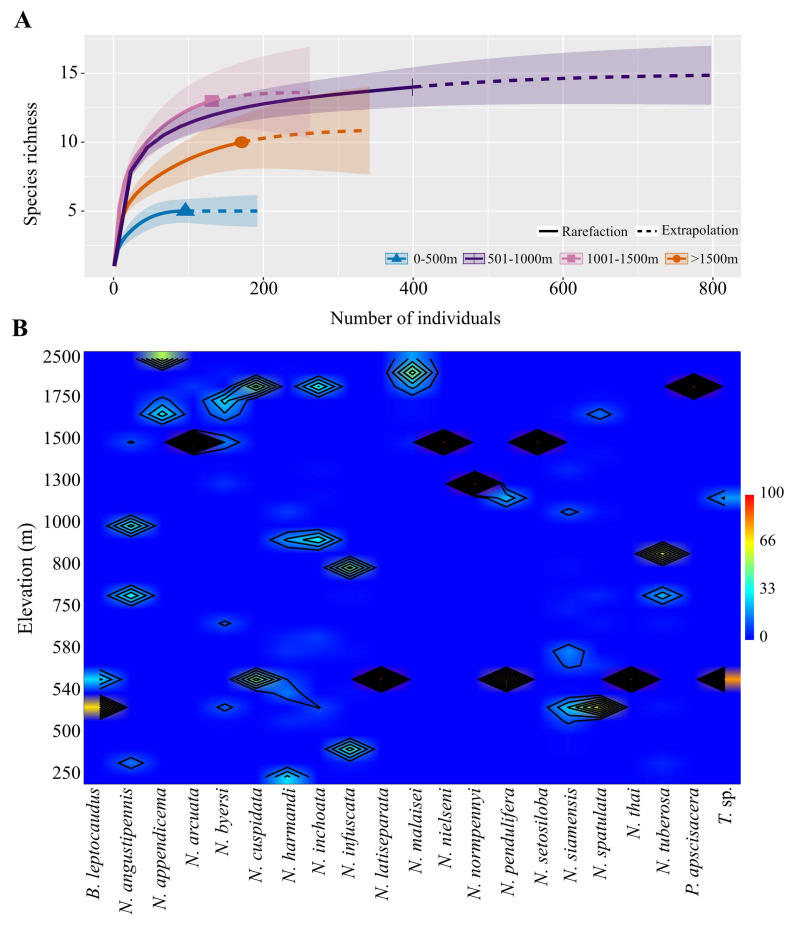
(**A**) Species accumulation curves (sample rarefaction based on abundance data) at different elevation zones, including 0–500 m, 501–1000 m, 1001–1500 m, and >1500 m. The shaded area represents 95% confidence intervals. Solid line = observed species (interpolation); dashed line = extrapolated species richness. Sample coverage of the reference sample for each elevation zones: 0–500 m = 1, 5001–1000 m = 0.995, 1001–1500 m = 0.985, >1500 m = 0.988, q = 0, species richness with bootstrap of 1000. (**B**) Elevational specific abundance (%) of mecopteran species.

Accumulation curve of an elevational species richness indicated that the middle elevations (501–1000 m asl) exhibited the highest species richness, while the lowest number of species were observed at low elevations (0–500 m asl). However, there was no significant difference in species number between the middle, high (1001–1500 m asl), and upper high elevation zones (>1500) (overlapping shaded regions) (Figure 5A). Mecopteran species richness distributed along the elevational gradient showed hump-shaped patterns, with an increasing number of species from low elevation (5 species) to the highest number at middle elevations (15 species), and then declining in the high and upper high elevations, where 13 and 10 species were found, respectively.

Elevational variation in mecopteran assemblages differed among species. Most species (nine species: *Neopanorpa angustipennis*, *N. byersi*, *N. cuspidata*, *N. harmandi*, *N. inchoata*, *N. pendulifera*, *N. siamensis*, *N. spatulata*, and *Terrobittacus* sp.) displayed a wide vertical distribution range from low (250 m) to high elevations (2200 m). Conversely, *N. appendicema*, *N. arcuata*, *N. malaisei*, *N. nielseni*, *N. normpennyi*, *N. setosiloba*, and *Panorpa apscisacera* were found only at elevations exceeding 1300 m. Furthermore, four species, *N. infuscata*, *N. latiseparata*, *N. thai*, and *N. tuberosa* were observed in areas at elevations lower than 800 m, while *Bittacus leptocaudus* was distributed between 500 m and 1200 m (Figure 5B).

### 3.3. Impact of Environmental Variables on Mecopteran Species Composition

**Figure 6:**
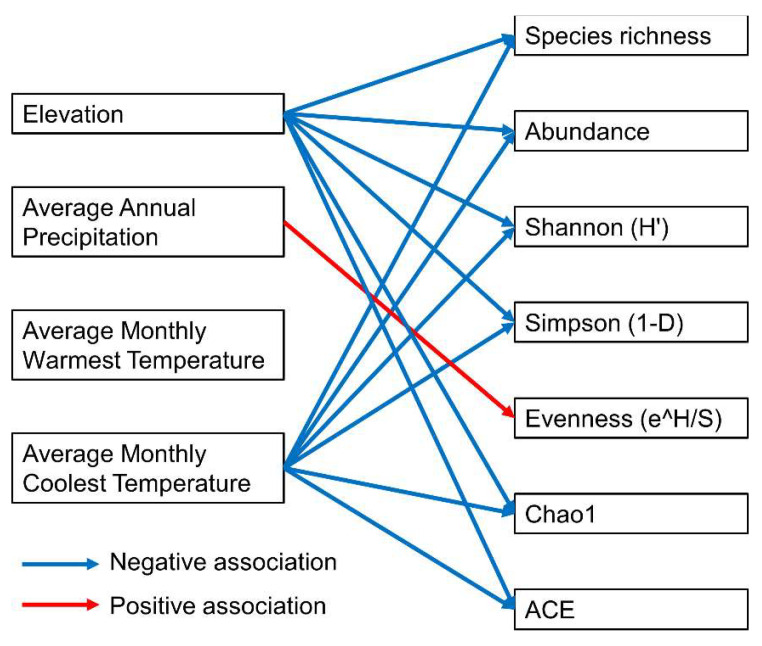
Summary of associations between diversity and abundance indices with environmental factors.

The relationships between various diversity and abundance indices and environmental factors are presented in Table 2. The average monthly coolest temperature (AMCT) and elevation were negatively correlated with species richness, abundance, Shannon (H′), Simpson (1-D), Chao1, and ACE (Table S1). For evenness (e^H/S), a positive correlation was noted with AAP (slope = 0.0002 ± 0.0001, t-value = 2.35, *p* < 0.05) (Table 2). The overall associations between diversity and abundance indices with environmental factors are summarized in Figure 6. These results supported the assumption that the evenness of mecopteran species increases with the increase in rainfall or humidity. Differently, species richness and abundance indices are negatively correlated with elevation and temperature (Figure 6), indicating that species richness decreases with the increase in temperature and elevation.

## 4. Discussion

This study focused on the small insect order Mecoptera in Thailand, providing more insights into their biodiversity and phenology. Twenty-one mecopteran species (four genera) were reported in this study, including one unidentified species of a newly recorded genus, *Terrobittacus*. This differed slightly from a previous report by Bicha in 2022 [3], which documented 22 species from three genera (*Bittacus*, *Neopanorpa* and *Panorpa*), thereby bringing the total mecopteran fauna in Thailand to 23 species. *Terrobittacus* is a small genus comprising eight species that are endemic to China and distributed only at high elevations (950–2500 m) with cool conditions and moist forests [45,46,47]. The current study found this genus during the rainy season at elevations of 567 m (four specimens) and 1231 m (one specimen). Although these locations are somewhat lower than those previously recorded in China, the conditions of habitat are similar to those with cool temperature and high humidity in the forest.

Thailand boasts a remarkable 22 mecopteran species, constituting 39% of the total species recorded from Indochina (56 species, including 48 in *Neopanorpa*, 3 in *Panorpa*, 4 in *Bittacus* and 1 in *Bicaubittacus*) [3], indicating a high biodiversity of this insect group in this country when compared to its neighbors. For example, Laos had only two recorded species, while Myanmar and Vietnam had 19 and 17, respectively [1,38,48,49]. Due to the complexity of the topography, rich natural resources, and various types of forest in Laos, Myanmar, Thailand and Vietnam, suitable habitats are provided for mecopteran diversity [50,51,52]. Considering species coexistence in Thailand, Myanmar, and Vietnam, only one species (*N. malaisei*) out of approximately 56 total species recorded in all three countries was found to co-occur. Furthermore, one species (*N. nielseni*) was identified only in Thailand and Vietnam, and two (*N. malaisei* and *N. angustipennis*) coexisted in Thailand and Myanmar [3,38,49]. There are two probable reasons for low species coexistence: first, high habitat specificity [9,10,53], and second, insufficient surveys in those countries. When comparing Thailand and China, five species were found to coexist: *N. harmandi*, *N. nielseni*, *N. cuspidata*, *N. siamensis* and *N. spatulata* [9,54]. This indicates that some species of Mecoptera have been widely dispersed. It is assumed that many new species of Mecoptera are undiscovered in unreachable areas in Thailand. Therefore, extensive entomological surveys are urgently required for a better understanding of the biodiversity of this insect order, especially in high mountains where these insects are known to prefer low temperatures and a well-balanced level of moisture throughout the growing season, due to its weak migration ability [9,10,55].

Information concerning the distribution and seasonal variation of Mecoptera has not been reported in several areas since their original description, and several species were previously known only from a lone specimen [1]. This study provides more details on geographical distribution of each Thai species and found that *Neopanorpa siamensis* is the most widely distributed species [56]. Consistent with the original species description, *N. siamensis* was collected from several areas at elevations ranging between 540 and 900 m, these mostly in the northern region, such as Fang district, Doi Chiang Dao and Doi Suthep in Chiang Mai province. Some specimens were collected at an elevation of 1260 m in Phu Kradung, Loei province, northeastern Thailand [37]. Moreover, this study provides more information on the elevational distribution of *N. siamensis*, which was found at between 107 and 1639 m with high abundance between 500 and 600 m.

In this study, several species were found in only one or two collection sites. This was likely due to inappropriate sampling methods and trapping locations for Mecoptera, or they had highly specific microhabitat requirements. For instance, *N. nielseni* could be found only in Doi Pha Hom Pok National Park at an elevation of 1449 m, while other reports showed a wide range of distribution for this species, which was common in Vietnam [1,37]. This species was recorded recently in several localities at elevations of between 1230 and 2000 m in Yunnan province, China [9]. Mecoptera are sometimes clearly the most abundant conspicuous insect in the right season and location [36]. Future studies on the distribution of mecopteran species correlating with environmental factors are crucial for their conservation and value as indicator species in assessing the effects of climate change or change in forest structure.

*Neopanorpa harmandi* is the most relatively abundant and the second most widely distributed species. This finding aligns with previous reports that this species occurred in multiple provinces in Thailand: Chiang Mai, Mae Hong Son, and Phrae in the northern region, Chonburi in the eastern region, Nakhon Ratchasima in the northeastern region, and Saraburi in the central region [36,37]. *N. harmandi* also has a wide geographical distribution, occurring in several Oriental countries, including Cambodia, Vietnam and China [1,9].

The estimated species richness was very high in this study, suggesting that there might be many new species still undiscovered. This trend is similar to that in several other insect groups which revealed different percentages of unrecorded species, such as 85% of longhorn beetle species [29] and 10–20% of Diptera and Auchenorrhyncha in the tropical forests of Thailand [31], as well as 44% of ant species in the Amazonian rainforest [57] and 80% of terrestrial insect communities in the riparian zone of the Miho River, Korea [58]. This study sampled specimens using passive methods (Malaise and pan traps), without baits or other attractants. This approach requires insects to fly into traps of their own accord, which is ineffective for Mecoptera, due to their limited dispersal abilities [10,55]. As a result, the collections yielded a limited number of specimens or species. In fact, the standard (most suitable or effective) methods for catching Mecoptera include sweep netting; direct searching in target habitats, using rotting squid as bait on the upper leaf surface of herbaceous plants at selected sites; yellow pan trap; green multi-funnel trap; flight interception trap; Malaise trap; and pyrethroid fogging [1,8,9,14,22,49,55,59,60]. These methods would be more effective for capturing greater numbers of specimens and additional species of Mecoptera. For instance, sweep netting in China caught approximately 20 species in the Hengduan mountains of Yunnan province [9], and 14 species (2662 individuals) of scorpionflies in the Qinling mountains of Shaanxi province [22]. Therefore, this study recommends employing multiple methods for collecting Mecoptera, which would provide more comprehensive and accurate results. However, the Malaise trap is advantageous in saving time and effort while still providing valuable information for long-term studies [8].

Information on the seasonal occurrence and distribution of Mecoptera in Southeast Asia has historically been limited to little more than the type series [5]. We found that species richness was highest in the rainy season. This trend is similar to the previously reported emergence patterns of most Thai species, which typically occur during the rainy to early cold seasons [5,36,37]. The peak of species richness during the rainy and cold seasons is mainly attributed to humidity and temperature, consistent with mecopteran diversity patterns reported previously [2,10,22]. These patterns also were observed in the Oriental region, such as Myanmar, where a few species were predominant during September and October (rainy season) [38]. The small population of *Austromerope poultoni* peaked in winter (June to August) in Australia and extended into spring (September to November) [61]. The Chilean, *Notiothauma reedi* [62], is another example of a mecopteran that is primarily active in the cool, moist autumn. Likewise, some species from France are abundant during May to August, with peak abundance varying depending on species [14]. *Neopanorpa harmandi* from Cambodia are active during May to August [1], while in Thailand, the species is active for a slightly longer period, extending from April into September [36,37]. *N. nielseni* in this study was found only in September and October, while a report from Yunnan province, China, said it occurred between April and August [9]. This difference may be related to the temporal shift between China and Thailand [14]. The soil is typically very moist during the rainy season, allowing the larvae to develop, and herbaceous plants and shrubs to grow. This is associated with adults found resting frequently on shrubs or herbaceous plants in moist forests [14,18]. This is in contrast to a report from south-west Western Australia where a primitive mecopteran, *Austromerope poultoni*, occurred throughout a wide range of rainfall and was not associated with plants, as its adults spend most of their time on the ground [61]. Another primitive ground-dwelling mecopteran, *Notiothauma reedi*, is primarily active in the autumn, feeding on decaying corpses [62,63].

The spatial distribution of insects has been reported in various insect groups, and the results revealed different relationships depending on the taxa [23,29,30,64,65,66,67]. Our result showed a hump-shaped distribution pattern of this insect, which was similar to previous reports from the Qinling mountains in Yunan province and the Sichuan Basin in Sichuan province, China, suggesting that the highest species numbers occurred in the middle elevation [22,68]. However, different patterns have been reported in the Caucasus in Georgia [55], where only three species of the genus *Panorpa* have been collected at altitudes ranging from 666 to 2559 m above sea level. These differences may be influenced by other factors such as microhabitat, topography, and collection methods [22]. Previous studies showed that elevation and precipitation are the main factors influencing species richness, with forest at intermediate elevations being identified as the most suitable habitat [10,22]. Furthermore, *Panorpa* typically inhabits high elevation moist forests (800–3000 m) in temperate and subtropical areas, like the *P. apscisacera* found in this study at 2112 m [15] and several species from Mexico were also recorded at elevations of between 3850 and 9600 feet [69,70]. As in this study, *Neopanorpa nielseni* was found at high elevations ranging between 1230 and 2000 m in China [9].

Previous research has demonstrated that members of other Mecoptera families exhibit cold resistance; notably, the snow scorpionflies of the Boreidae family [71] were reported to exhibit such adaptations. This family is recognized for its ability to regulate trehalose and glycerol concentrations in their hemolymph as a strategy to endure low-temperature environments [71]. *Notiothauma reedi* (Eomeropidae), *Austromerope poultoni* (Meropidae) and Tasmanian snow scorpionflies within the Apteropanorpidae [72], are also active in the cool season. These insects are phylogenetically proximate to the Panorpidae [7], suggesting that the capacity for cold resistance may be an ancestral trait within these lineages. This inference is supported by observations of cold resistance in numerous Panorpidae species [10,53]. Despite the presence of Panorpidae in tropical and subtropical regions [10], phylogenetic analyses and species distribution modeling for certain species, such as *Dicerapanorpa magna* (family: Panorpidae), suggest that their contemporary distribution patterns were established during the Pleistocene epoch, accompanied by significant habitat fragmentation. This species, in particular, exhibits a highly specialized distribution, confined to isolated mountainous regions [73].

The observed inverse relationship between the diversity of Mecoptera and temperature further suggests a process of niche differentiation among species. Thornhill [74] posited that interspecific competition among Panorpidae has led to the differentiation of niches based on vertical distribution. This results in more aggressive species occupying lower vertical strata, where conditions are presumably more favorable, while less aggressive species are relegated to higher strata, characterized by less favorable conditions [74]. Both *Neopanorpa* and *Panorpa* larvae are euedaphic, living in the soil most of their life time. However, *Panorpa* larvae emerge to the surface, likely to forage at night, to a significantly greater degree than *Neopanorpa* [75]. We speculate that *Panorpa* larvae would have a much greater opportunity for survival at higher elevations and also during cooler seasons with less competition for food as well as less predation, where, and when, ants are less abundant. To validate this hypothesis, further research is needed to investigate the correlation between the aggressiveness of Panorpidae and their environmental preferences.

The observed positive correlation between humidity levels and species evenness within the Mecoptera order suggests that increased environmental humidity facilitates a broader niche for less common species. This expansion in niche availability for rarer species leads to a reduction in the relative abundance of dominant species within the community. This phenomenon is hypothesized to be linked to the biological characteristics of the larval stage in Panorpidae scorpionflies. Specifically, Panorpidae are known to deposit their eggs in moist soil [18], and their larvae exhibit reduced sclerotization along with specialized morphological traits [75], adaptations that are conducive to surviving and foraging within humid soil environments. Similar adaptations, associated with diminished desiccation tolerance, have been observed in other insects, such as termites [76]. We propose that the observed increase in community evenness is a direct consequence of higher humidity levels providing a more abundant habitat, thereby supporting the oviposition and larval foraging activities of Panorpidae.

This study provides an overview and detailed insights into mecopteran diversity and its community structure, including their spatiotemporal variation and geographical distribution in Thailand. The results derived from multiple regression analyses indicate a negative association between species richness and both temperature and altitude. This suggests a lower likelihood of Mecoptera diversity in regions characterized by elevated temperatures and high altitudes. Such a trend elucidates the observed patterns of Mecoptera diversity becoming reduced in low and high elevation areas. We suggest that both the elevated temperatures in lowland areas and the extreme conditions at high altitudes appear to impose constraints on the distribution of Mecoptera.

## 5. Conclusions

This study represents the first comprehensive report on the biodiversity, ecology, and geographical distribution of Mecoptera in Thailand. A total of 21 species (4 genera) were found, including the first-time report of the hangingfly in the genus *Terrobittacus* in the country. *Neopanorpa harmandi* was the most relatively abundant species, while *N. siamensis* exhibited the widest distribution. High species diversity was observed at Doi Pha Hom Pok and Umphang. Interestingly, 51% of the total species were specific to certain regions. Seasonal species richness is higher in the rainy season than the cold and hot seasons. The influences of elevation and climatic factors on species composition are evident, with mid-altitude areas being important for species diversity. Additionally, species richness and abundance indices are negatively correlated with elevation and temperature. For the efficient collection of Mecoptera, various collection methods are recommended, with sweep netting being the most suitable one. However, using Malaise and pan traps, which are cost-effective and require less human effort still provide sufficient valuable baseline data for future studies.

## Figures and Tables

**Table 1:** Species composition and abundance of Mecoptera in Thailand, collected from 2006 to 2009 and 2020 to 2021.

National Parks	Locality Name	Elevation (m, msl)	*B. leptocaudus*	*N. angustipennis*	*N. appendicema*	*N. arcuata*	*N. byersi*	*N. cuspidata*	*N. harmandi*	*N. inchoata*	*N. infuscata*	*N. latiseparata*	*N. malaisei*	*N. nielseni*	*N. normpennyi*	*N. pendulifera*	*N. setosiloba*	*N. siamensis*	*N. spatulata*	*N. thai*	*N. tuberosa*	*P. apscisacera*	*T*. sp.	No. of Specimens	Species Richness/Sites
Doi Pha Hom Pok	Mae Fang Hotspring	569							3									10	4					17	3
	Doi Pha Luang	1449		2		28	14						3	6			15	1						69	7
	Kiewlom	2112				2	1	1		19			15									3		41	6
Doi Inthanon	Vachiratharn	700					9												2		1			12	3
	Checkpoint 2	1639			8		14						1					2	8					33	5
	Kew Mae Pan	2200					2		1				35											38	3
	Summit	2500			14								15											29	2
Doi Chiang Dao	Pha Tang substation	526	5				10			5								25	38		1			84	6
Huai Nam Dang	Headquarters	1670			3		25						1						1					30	4
Doi Phu Kha	Headquarters	1374					1			1								4						6	3
Chae Son	Waterfall	507					1		12	2								10						25	4
	Doi Lan	1413																1	1					2	2
Phu Ruea	Rong Huay Maklaow	1167							18									11	1					30	3
Pa Hin Ngam	Tung Dok Grajeaw	780							9	4														13	2
Khao Yai	Headquarters	770		7							1							2	3		9			22	5
Nam Nao	Checkpoint	921							43	18														61	2
	Sam Makao forest unit	528							34		1							1						36	3
Khao Kho	Headquarters	230							66												1			67	2
Thung Salaeng Luang	Gang Wang Nam Yen	580							14	2	1							16						33	4
Mae Wong	Chong Yen	1306					5								16									21	2
Khao Khitchakut	Prabaht unit	107																1						1	1
	Khao Prabaht peak	875																			24			24	1
Umphang	Mae Klong Kee	1231								1						1							1	3	3
	Thi Lor Su	567	2					1	19			1				3				5			4	35	7
Khuean Srinagarindra	Tham Nanya	750																3						3	1
Kaeng Krachan	Panern Thung	790									24							2						26	2
	Pa La-U waterfall	320									20							1						21	2
Namtok Yong	Protection unit 3	372		3							2										2			7	3
	TV aerial	952		8																				8	1
No. of specimens (*n* = 797)			7	20	25	30	82	2	219	52	49	1	70	6	16	4	15	90	58	5	38	3	5	797	
Relative abundance (%RA)			0.9	2.6	3.1	3.8	10.3	0.2	27.4	6.5	6.1	0.1	8.8	0.8	2.0	0.5	1.9	11.3	7.3	0.6	4.8	0.4	0.6		
No. of positive sites (*n* = 29)			2	4	3	2	10	2	10	8	6	1	6	1	1	2	1	15	8	1	6	1	2		
Species occurrence (% SO)			6.9	13.8	10.3	6.9	34.5	6.9	34.5	27.6	20.7	3.4	20.7	3.4	3.4	6.9	3.4	51.7	27.6	3.4	20.7	3.4	6.9		
Regional distribution *			N, W	N, S, NE	N	N	N, C	N, W	N, C, W, N, E	N, C, NE, W	NE, C, W, S	W	N	N	C	W	N	N, C, NE, E, W	N, NE	W	N, NE, C, E, S	N	W		

Abbreviations: * N = northern, E = eastern, NE = northeastern, C = central, W = western, and S = southern.

**Table 2:** Relationships between diversity and abundance indices with environmental factors. This table presents the associations of diversity or abundance indices with environmental variables: elevation (range: 107–2500), average annual precipitation (AAP) (range: 1064–2503), average monthly warmest temperature (AMWT) (range: 22–32), and average monthly coolest temperature (AMCT) (range: 12–22). Analyses were conducted using multiple regression models, and stepwise selection was employed to exclude variables that were not statistically significant.

Dependent Variable (Value Range): Formula	Formula	Model R^2^	Statistics of Independent Variable			
			Independent ^a^ Variable	Slope ^b^	t-Value	*p* Value
Species richness (1–7)	Species richness = 19. 8343 −0.0028 × Elevation −0.7836 × AMCT	0.38	Intercept	19. 8343 ± 4.4557	4.45	<0.001
			Elevation	−0.0028 ± 0.0009	−2.92	<0.01
			AMCT	−0.7836 ± 0.2023	−3.87	<0.001
Abundance (1–84)	Abundance = 182.1138 −0.0275 × Elevation −7.1669 × AMCT	0.20	Intercept	182.1138 ± 62.0492	2.94	<0.01
			Elevation	−0.0275 ± 0.0132	−2.09	<0.05
			AMCT	−7.1669 ± 2.8178	−2.54	<0.05
Shannon (H′)	Shannon (H′) = 5.5581 −0.0008 × Elevation −0.2266 × AMCT	0.37	Intercept	5.5581 ± 1.3554	4.10	<0.001
(0–1.578)			Elevation	−0.0008 ± 0.0003	−2.65	<0.05
			AMCT	−0.2266 ± 0.0616	−3.68	<0.01
Simpson (1-D)	Simpson (1-D) = 2.8059 −0.0003 × Elevation −0.1134 × AMCT	0.30	Intercept	2.8059 ± 0.8437	3.33	<0.01
(0–1)			Elevation	−0.0003 ± 0.0002	−1.93	0.06
			AMCT	−0.1134 ± 0.0383	−2.96	<0.01
Evenness (e^H/S) (0.441–1.396)	Evenness (e^H/S) = 0.4599 +0.0002 × AAP	0.17	Intercept	0.4599 ± 0.1611	2.86	<0.01
			AAP	0.0002 ± 0.0001	2.35	<0.05
Chao1 (1.000–7.486)	Chao1 = 20.6737 −0.0028 × Elevation −0.8189 × AMCT	0.38	Intercept	20.6737 ± 4.7513	4.35	<0.001
			Elevation	−0.0028 ± 0.0010	−2.74	<0.05
			AMCT	−0.8189 ± 0.2158	−3.80	<0.001
ACE (1.000–8.184)	ACE = 23.0892 −0.0028 × Elevation −0.9252 × AMCT	0.35	Intercept	23.0892 ± 6.1352	3.76	<0.001
			Elevation	−0.0028 ± 0.0013	−2.14	<0.05
			AMCT	−0.9252 ± 0.2786	−3.32	<0.01

^a^ AMWT was excluded from all models during the step-wise model selection process. ^b^ The diminutive slope values for elevation and AAP (<0.01) can be attributed to the extensive scales of their respective value ranges—elevation spanning 107 to 2500 and AAP ranging from 1064 to 2503. This contrasts with the narrower ranges of the dependent variables, such as Shannon’s diversity index (H′) (0 to 1.578), Simpson’s diversity index (1-D) (0 to 1), and evenness (e^H/S) (0.441 to 0.136). Despite their minimal magnitude, these slopes effectively predict the dependent variables’ ranges within the models’ equations.

## Data Availability

The data presented in this study are available in the Supplementary Materials.

## Supplementary Materials

The following supporting information can be downloaded at: https://www.mdpi.com/article/10.3390/insects15030151/s1, Table S1: Collection sites, biodiversity data and climatic factors of Mecoptera in Thailand.
