# Supplementary material for: Biodiversity and Spatiotemporal Variations of Mecoptera in Thailand: Influences of Elevation and Climatic Factors
Source: Insects. 2024 Feb 23;15(3):151. doi: 10.3390/insects15030151 (PMC10971531; doi:10.3390/insects15030151)
Supplement: Supplementary file 1 [file insects-15-00151-s001.zip › insects-2865980-supplementary.pdf]

Table S1. Collection sites, biodiversity data and climatic factors of Mecoptera in Thailand.

National park	Site name	Locality code	Region	Geographic coordinate	Elevation (m, msl)	Diversity indices						Climatic factors*		
						Species richness	Individuals	Shannon _H	Simpson _1-D	Evenness _e^H/S	Chao-1	AAP	AMWT	AMCT
Doi Pha Hom Pok	Mae Fang Hotspring	1	North	19°57.961'N 99°9.355'E	569	3	17	1.02	0.60	0.92	72	1357.70	28.63	18.10
	Doi Pha Luang	2	North	20°1.06'N 99°9.581'E	1449	7	69	1.58	0.75	0.69		1356.68	26.17	15.54
	Kiewlom	3	North	20°3.549'N 99°8.553'E	2112	6	41	1.31	0.66	0.61		1355.63	23.40	12.79
Doi Inthanon	Vachiratharn	4	North	18°32.311'N 98°36.048'E	700	3	12	0.80	0.44	0.75	25	1125.33	28.58	17.79
	Checkpoint 2	5	North	18°31.559'N 98°29.941'E	1639	5	33	1.39	0.72	0.80		1173.82	25.60	14.75
	Kew Mae Pan	6	North	18°33.162'N 98°28.81'E	2200	3	38	0.35	0.15	0.47		1234.43	22.60	11.85
	Summit	7	North	18°36.361'N 98°29.157'E	2500	2	29	0.71	0.52	1.02		1235.18	22.35	11.75
Doi Chiang Dao	Pha Tang substation	8	North	19°24.978'N 98°54.886'E	526	6	84	1.39	0.69	0.67	19	1242.15	26.58	16.17
Huai Nam Dang	Headquarter	9	North	19°18.712'N 98°36.399'E	1670	4	30	0.66	0.30	0.48	9	1222.80	25.44	15.04
Doi Phu Kha	Headquarter	10	North	19°12.562'N 101°4.953'E	1374	3	6	1.03	0.60	0.94	5	1292.75	28.60	17.25
Chae Son	Waterfall	11	North	18°50.172'N 99°28.38'E	507	4	25	1.11	0.63	0.76	13	1205.45	29.67	18.71
	Doi Lan	12	North	18°51.815'N 99°22.122'E	1413	2	2	0.94	1.00	1.28		1192.45	26.83	16.17
Phu Ruea	Rong Huay Maklaow	13	Northeast	17°29.652'N 101°21.02'E	1167	3	30	0.82	0.52	0.76	5	1297.72	29.02	17.83
Pa Hin Ngam	Tung Dok Grajeaw	14	Northeast	15°38.438'N 101°23.576'E	780	2	13	0.66	0.46	0.96	3	1236.35	29.85	19.29
Khao Yai	Headquarter	15	Northeast	14°24.781'N 101°22.689'E	770	5	22	1.45	0.74	0.85	13	1248.45	28.00	18.35
Nam Nao	Checkpoint	16	Central	16°43.687'N 101°33.754'E	921	2	61	0.61	0.42	0.92	9	1205.15	28.17	17.63
	Sam Makao forest unit	17	Central	16°41.067'N 101°40.425'E	528	3	36	0.28	0.11	0.44		1199.23	29.90	19.35
Khao Kho	Headquarter	18	Central	16°39.550'N 101°08.123'E	230	2	67	0.09	0.03	0.54	3	1321.62	32.33	21.27
Thung Salaeng Luang	Gang wang Nam Yen	19	Central	16°34.57'N 100°53.16'E	580	4	33	1.04	0.60	0.70	9	1256.88	29.17	18.25
Mae Wong	Chong Yen	20	Central	16°5.212'N 99°6.576'E	1306	2	21	0.57	0.38	0.89	3	1555.53	27.02	16.17
Khao Khitchakut	Prabaht unit	21	East	12°48.779'N 102°9.181'E	107	1	1	–	–	–	3	2502.58	30.75	22.38
	Khao Prabaht peak	22	East	12°50.43'N 102°9.83'E	875	1	24	–	–	–		2186.67	28.71	20.29

National park	Site name	Locality code	Region	Geographic coordinate	Elevation (m, msl)	Diversity indices						Climatic factors*		
						Species richness	Individuals	Shannon _H	Simpson _1-D	Evenness _e^H/S	Chao-1	AAP	AMWT	AMCT
Umphang	Mae Klong Kee	23	West	16°13'30.8"N 98°58'47.1"E	1231	3	3	1.43	1.00	1.40	33	1617.20	26.90	15.96
	Thi Lor Su	24	West	15°55'14.5"N 98°45'53.0"E	567	7	35	1.52	0.68	0.65		1961.45	29.13	18.23
Khuean Srinagarindra	Tham Nanya	25	West	14°41.604'N 98°53.035'E	750	1	3	–	–	–	1	1968.45	29.42	18.71
Kaeng Krachan	Panern Thung	26	West	12°47.831'N 99°27.369'E	790	2	26	0.29	0.15	0.67	3	1063.95	28.96	20.96
	Pa La-U waterfall	27	West	12°32.154'N 99°28.098'E	320	2	21	0.22	0.10	0.62		1131.83	29.13	21.46
Namtok Yong	Protection unit3	28	South	8°13.434'N 99°48.295'E	372	3	7	1.22	0.76	1.13	5	2045.58	29.67	21.31
	TV aerial	29	South	8°14.262'N 99°48.289'E	952	1	8	–	–	–		2045.58	29.67	21.31

Abbreviations: * AAP, Average Annual Precipitation; AMWT, Average Monthly Warmest Temperature; AMCT, Average Monthly Coolest Temperature.
